# Epitaxial intergrowths and local oxide relaxations in natural bixbyite Fe_2−*x*_Mn_*x*_O_3_

**DOI:** 10.1107/S2052252522006315

**Published:** 2022-06-29

**Authors:** Kristoffer Andreas Holm Støckler, Nikolaj Roth, Thomas Bjørn Egede Grønbech, Bo Brummerstedt Iversen

**Affiliations:** a Center for Integrated Materials Research, Deparetment of Chemistry and iNANOAarhus University, Langelandsgade 140, Aarhus 8000, Denmark

**Keywords:** 3D-ΔPDF, diffuse X-ray scattering, diffuse neutron scattering, bixbyite, defects

## Abstract

The scattering pattern of a crystal obeys the symmetry of the crystal structure through the corresponding Laue group. This is usually also true for the diffuse scattering, containing information about disorder, but here a case is reported where the diffuse scattering is of lower symmetry than the parent crystal structure. The mineral bixbyite has been studied by X-ray and neutron scattering techniques since 1928 with some of the most recent studies characterizing the low-temperature transition to a magnetically disordered spin-glass state. However, bixbyite also exhibits structural disorder, and here single-crystal X-ray and neutron scattering is used to characterize the different modes of disorder present. One-dimensional rods of diffuse scattering are observed in the cubic mineral bixbyite, which break the expected symmetry of the scattering pattern. It is shown that this scattering arises from epitaxial intergrowths of the related mineral, braunite. The presence of this disorder mode is found to be directly observable as well-defined residuals in the average structure refined against the Bragg diffraction. An additional three-dimensional diffuse scattering component is observed in neutron scattering data, which is shown to originate from the substitutional disorder on the Fe/Mn sites. This occupational disorder gives rise to local relaxations of the oxide sublattice, and the pattern of oxide displacements can be rationalized based on crystal-field theory. The combined use of neutron and X-ray single-crystal scattering techniques highlights their great complementarity. In particular, the large sample requirements for neutron scattering experiments prove to be an obstacle in solving the intergrowth disorder due to several growth orientations, whereas for X-ray scattering the one-dimensional nature of the intergrowth disorder renders solving this a more tractable task. On the other hand, the oxide relaxations cannot be resolved using X-rays due to the low Mn/Fe contrast. By combining the two approaches both types of disorder have been characterized.

## Abstract 2

Different types of structural disorder in natural bixbyite are quantified from analysis of X-ray and neutron diffuse scattering

## 1. Introduction

Crystal intergrowth structures pose a great challenge for structural studies using traditional characterization methods such as single-crystal or powder X-ray Bragg diffraction due to the discrepancy between the local structure and the average crystallographic structure (Rao, 1985 ▸). Thus, local probes such as high-resolution transmission electron microscopy (HR-TEM) and integrated differential phase contrast scanning transmission electron microscopy (iDPC-STEM) have been used successfully to visualize the two-dimensional projection of the atomic structure at the solid–solid interface of the intergrowth structures in materials such as intergrowth forming minerals and industrially important zeolites (Shen *et al.*, 2021 ▸; Kleebe & Lauterbach, 2008 ▸; Peter *et al.*, 2021 ▸; Devilliers & Buseck, 1989 ▸; Moore & Araki, 1976 ▸). However, single-crystal diffuse scattering offers a way of probing how the local microscopic structure deviates from the average structure over a macroscopic three-dimensional sample. Thus, the experimental information extracted from this can be used to generalize the local structure information extracted from the TEM measurements. Furthermore, single-crystal diffuse scattering methods are sensitive to three-dimensional disorder/local order, which can be difficult to quantify using microscopy techniques where only a two-dimensional projection of the sample is analyzed. This includes small atomic displacements, which can be difficult to resolve using TEM. Here, we show how single-crystal X-ray and neutron scattering can be used to solve the structure of disordered intergrowths and local oxide relaxations in natural bixbyite crystals. It is shown how the cubic mineral bixbyite contains epitaxial intergrowth layers of the mineral braunite.

**Figure 1:**
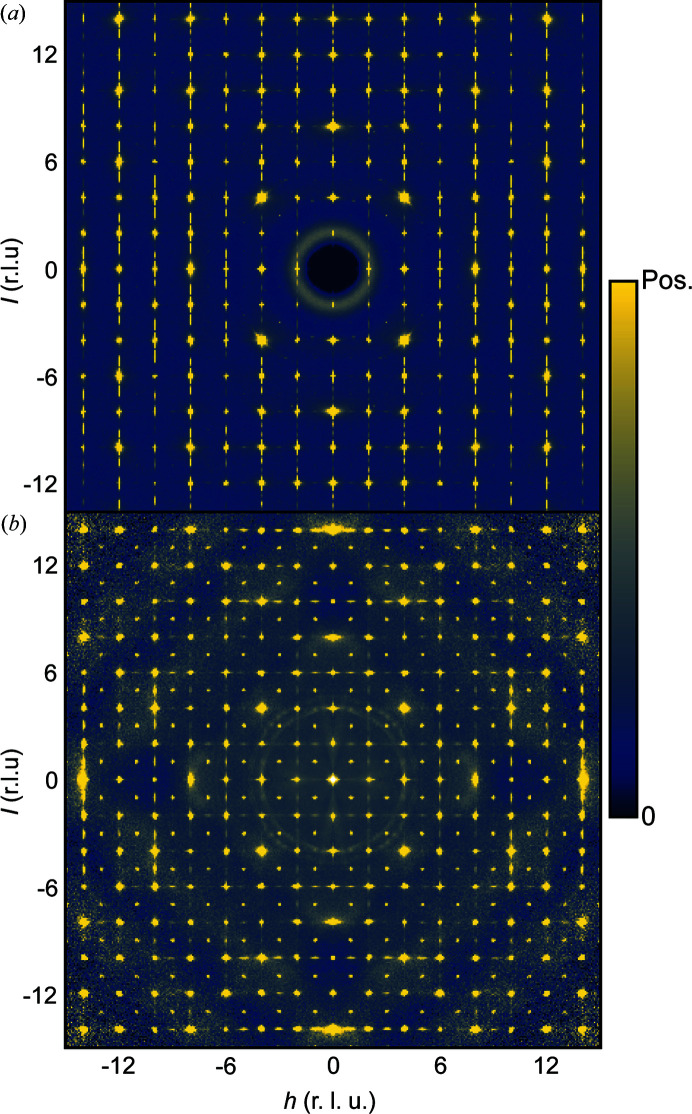
Experimental single-crystal (*a*) X-ray and (*b*) neutron scattering in the *h*0*l* plane. Distances in reciprocal space are given in reciprocal lattice units (r.l.u.). Both scattering patterns include Bragg and diffuse scattering. The data are shown on an arbitrary scale chosen to highlight the diffuse scattering features.

The structure of the iron-manganese oxide mineral bixbyite (Fe_2−*x*_Mn_*x*_O_3_, cubic, *Ia* 3, *a* = 9.41 Å) has been studied by scattering methods (X-ray and later neutron scattering) since 1928 (Zachariasen, 1928 ▸; Patterson, 1939 ▸). Most recently, novel methods based on the analysis of single-crystal magnetic neutron diffuse scattering have been used to study the low-temperature magnetic structure of the material, which displays short-range spin-glass like correlations (Roth *et al.*, 2019 ▸, 2018 ▸). However, the naturally occurring mineral also contains interesting structural disorder, which has not previously been studied in detail. This structural disorder gives rise to diffuse scattering, shown in Fig. 1 ▸ for both X-ray and neutron scattering.

In Fig. 1 ▸(*a*) single-crystal X-ray scattering (SCXRS) data from bixbyite is shown in the *h*0*l* plane of reciprocal space. Sharp rods of diffuse scattering with intensity modulations are observed along the c* direction indicating the presence of two-dimensional order/one-dimensional disorder. The dimensionality of the disorder and thus the diffuse scattering results in this material being an example where the diffuse scattering is of lower symmetry than the Laue group of the parent crystal structure. Previously, it has been established that naturally occurring bixbyite crystals from the Thomas Mountain Range in Utah, USA, where the crystals considered here originate from, exhibit lamellar epitaxial intergrowths of the closely related mineral braunite [Mn_7_SiO_12_, tetragonal, *I*4_1_/*acd*, *a* = 9.408 Å, *c* = 18.668 Å (Moore & Araki, 1976 ▸; Devilliers & Buseck, 1989 ▸; Peter *et al.*, 2021 ▸; Kleebe & Lauterbach, 2008 ▸)]. We will show that the diffuse rods in the SCXRS originate from such epitaxial intergrowths. As is also apparent from Fig. 1 ▸(*a*), additional diffuse scattering features reside in the vicinity of the Bragg peaks. The position of this diffuse scattering along with its characteristic shape hints at its origin being correlated atomic displacements, *i.e.* the diffuse scattering can be classified as thermal diffuse scattering (TDS).

In Fig. 1 ▸(*b*) single-crystal neutron scattering (SCNS) data are shown in the same plane of reciprocal space. By visual inspection, it can be seen that the diffuse rods observed in the SCXRS are still present, although they differ in several important ways. First, the relative intensities of the peaks of the modulated diffuse rods and the Bragg peaks are different compared with the SCXRS data. This is a result of the differences in element contrast. Second, the diffuse rods are observed along a*, b* and c*, which indicate the presence of braunite intergrowths along these directions, a consequence of the larger crystal size needed for the neutron scattering experiment. It is therefore particularly advantageous to use SCXRS to characterize the epitaxial intergrowths, as it is possible to measure data on a crystal small enough that it only has one distinct orientation of the intergrowth stacking, rendering the modelling of the disorder much less complicated.

In addition to the diffuse rods, broad three-dimensional diffuse scattering is also observed in the neutron data, see Fig. 1 ▸(*b*). As will be shown, this diffuse scattering is an effect of Fe and Mn sharing the same sites in the structure and, as opposed to the X-ray data, it can be resolved due to the higher element contrast. The X-ray scattering power of Fe and Mn can be determined by the number of electrons, which means that the contrast between the two elements is very low. Thus, any local order for which the metal ion identity is a determining factor will be highly challenging to investigate using X-rays. For this reason, the SCNS data are useful, since the scattering lengths of Fe (9.5 fm) and Mn (−3.7 fm) provide significant element contrast. Thus, the neutron data prove essential for the solution of the local structure.

## 2. Experimental

Single-crystal X-ray diffuse scattering data were collected at the NS ChemMatCARS 15-ID-D beamline at the Advanced Photon Source. A cubic (dimensions of ∼100 µm) single crystal of bixbyite was mounted at the end of a thin glass pin using ep­oxy glue. Data were collected using a Dectris Pilatus 1M CdTe detector at a sample-to-detector distance of 90 mm using 40 keV X-rays. Three different runs were collected with different in-plane detector off-sets in order to compensate for missing data due to gaps between detector modules. Each run consisted of a 360° omega scan, each frame spanning Δω = 0.1° with an exposure time of 1 s. After data collection, the crystal was removed and air scattering was collected using the same experimental parameters for later subtraction. The diffuse scattering data were reconstructed on a 701 × 701 × 701 voxel reciprocal space grid spanning ±35 in *h*, *k* and *l* (*Q*_max_ = 23.4 Å^−1^) using custom *MATLAB* scripts. During this process the averaged air scattering was subtracted and the data were corrected for polarization and a solid-angle correction was applied. The reconstructed data were symmetrized according to the *mmm* point group (a subgroup of both the *m* 3 Laue group of bixbyite and the 4/*mmm* Laue group of braunite) as this was found to be consistent with the symmetry of the diffuse scattering. The Bragg peaks were punched and filled using interpolated values as described by Holm *et al.* (2020 ▸). The isolated diffuse scattering was Fourier transformed in order to obtain the three-dimensional difference pair distribution function (3D-ΔPDF).

Single-crystal X-ray diffraction data were collected at the same beamline after diffuse scattering data collection. The experimental parameters were identical, except for the exposure time, which was limited to 0.1 s. The data were integrated using the *APEX3* software (Adam *et al.*, 2015 ▸). An empirical absorption correction was applied using *SADABS* (Krause *et al.*, 2015 ▸) while merging was performed in *SORTAV* (Blessing, 1997 ▸). The structure was solved and refined using *SHELXT* and *SHELXL* in the *OLEX2* GUI (Sheldrick, 2015*a* ▸,*b* ▸; Dolomanov *et al.*, 2009 ▸). In the final steps of the refinement, extinction was included as this was found to improve the agreement factor (*R*_1_) from 2.51 to 1.61%. This is mainly expected to be due to flux effects rather than due to actual extinction effects as discussed by Krause *et al.* (2020 ▸).

Single-crystal elastic neutron scattering was measured at the CORELLI instrument at the Spallation Neutron Source at Oakridge National Laboratory (Rosenkranz & Osborn, 2008 ▸; Ye *et al.*, 2018 ▸) on a piece cut from a larger bixbyite single crystal from the Thomas Range, Juab County, Utah, USA. Prior to the scattering experiment, the sample was sanded down to a sphere to limit crystal shape effects. The data used here were measured at 300 K, that is, in the paramagnetic regime. The data are the same as the 300 K data used for high-temperature subtraction in a previous study of the magnetic structure of bixbyite (Roth *et al.*, 2019 ▸).

## 3. Analysis and discussion

From the experimental scattering measurements both the Bragg scattering and the diffuse scattering are used to obtain a detailed picture of the atomic structure of bixbyite. From the Bragg peaks the average crystal structure can be solved, while the diffuse scattering contains information about the deviations from this average, such as the epitaxial intergrowths and the local atomic structure related to the Mn/Fe disorder. Models are developed for this disorder and compared with the experiment by calculating the diffuse scattering patterns using the *SCATTY* software (Paddison, 2019 ▸). For the X-ray scattering, the diffuse scattering is further Fourier transformed in order to obtain the 3D-ΔPDF. This function contains positive/negative features whenever the corresponding interatomic vector tends to separate more/less electron density in the real structure compared with the average structure (Weber & Simonov, 2012 ▸). Thus, it is a more intuitive function to interpret than the diffuse scattering function, which is given in reciprocal space. However, contrary to previous work, *e.g.* on Cu_2_Se (Roth & Iversen, 2019 ▸), the 3D-ΔPDF will not be used as a tool for building the supercell models directly but rather as a tool for validation of the model against data in real space.

### 3.1. Bixbyite average structure

**Figure 2:**
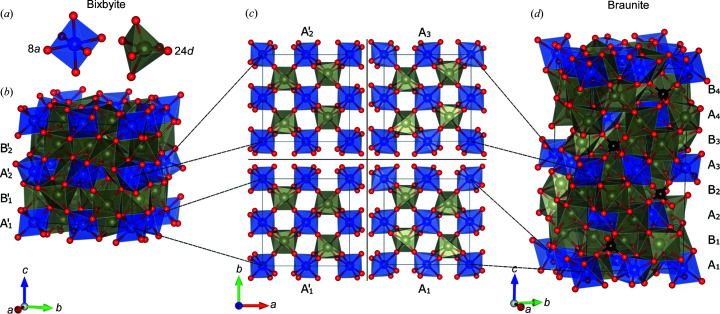
Crystal structures of (*b*) bixbyite and (*d*) braunite with the cation layers labelled as described in the text. In (*a*) the 8*a* site of bixbyite is coloured blue, while the 24*d* site is coloured grey. In (*c*) the corresponding sites in the braunite structure have been coloured equivalently to ease comparison of the (i) 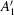 and *A*_1_ layers, and (ii) 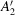 and *A*_3_ layers. The presence of silicon in the braunite *B_i_* is indicated by black spheres in (*d*).

The average structure of bixbyite crystals from the same location was reported by Roth *et al.* (2019 ▸, 2018 ▸) where the occupancies of Mn on the 24*d* and 8*a* sites, shown in Fig. 2 ▸(*a*), were refined to 0.509 (3) and 0.241 (5), respectively, with the Fe occupancy given as one minus the Mn occupancy. However, due to the low contrast between Fe and Mn in our X-ray experiments, we chose to only use Fe on the 24*d* and 8*a* sites and refine the occupancies. This results in a more stable refinement compared with including both Fe and Mn and refining their occupancies, and it gives a better description of the electron density compared with fixing the occupancies to the values found by neutron scattering. The contribution from Mn is thus effectively modelled as a slightly lower Fe occupancy. It was found that large electron density residuals resided near the 24*d* site, which resulted in large agreement factors. By placing an Fe atom at the location of the residual and constraining the occupancy of this atom and the Fe at the 24*d* site to add up to one, the agreement between data and model increased substantially. Although this average structure was at first only a convenient model for describing the electron density (particularly regarding the decision on leaving out Mn), we demonstrate in the following section that the presence of a large residual density near the original 24*d* site makes physical sense. We find that it can be attributed directly to the presence of lamellar braunite intergrowths in the bixbyite matrix.

### 3.2. Braunite intergrowth disorder

The bixbyite structure, shown in Fig. 2 ▸(*b*), can be considered to be built from two distinct types of layers along the *c* axis, the 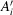 layers and the 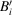 layers (*i* = 1, 2). Within the 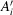 group the individual types are related by translations of (1/2, 1/2, 1/2) and similarly for the two layers of the 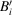 group. The 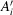 group, shown in Fig. 2 ▸(*c*), consists of distorted Fe/Mn—O octahedra for both the 24*d* and the 8*a* sites, while the 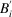 layers are exclusively made up by the distorted 24*d* site octahedra. The braunite structure, shown in Fig. 2 ▸(*d*), is closely related to the bixbyite structure, with unit cell parameters approximately corresponding to stacking two bixbyite unit cells along the *c* direction. In this structure, two distinct types of layers may also be defined. The first type labelled *A_i_* (*i* = 1, 2, 3, 4) contains layers which are isomorphic to the 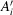 layers of bixbyite (Moore & Araki, 1976 ▸). By shifting the choice of origin in the braunite system by (1/2, 0, 0), the *A*_1_ layer of braunite is observed to be very closely related to the 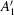 layer of bixbyite with the same being the case for the *A*_3_ and 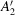 layers. The layers are shown in Fig. 2 ▸(*c*) for comparison. However, the isomorphic relations between the remaining *A_i_* layers of braunite and those of bixbyite are not as simple. The *A*_2_ layer of braunite may be produced from the *A*_1_ layer by first applying the mirror operation [0, 1, 0; 1, 0, 0; 0, 0, 1] and then displacing the origin of the unit cell by (1/4, 1/4, 0). Similarly the *A*_4_ layer may be produced by applying the same mirror operation and displacing the origin by (3/4, 3/4, 0). As will be described later, the mirror operation included in these isomorphic relations may be the mechanism responsible for the mirror twin reported in earlier neutron scattering studies (Roth *et al.*, 2019 ▸, 2018 ▸). While the 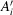 and *A_i_* layers are isomorphic, the 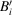 and *B_i_* layers are not. In particular, the *B_i_* layers of braunite contain Si, shown in Fig. 2 ▸(*d*), and therefore the intergrowths are clearly not of a polytypic origin. The 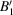 , *B*_1_ and *B*_3_ layers are shown explicitly in Fig. 5(*c*).

In the following, we show that the 1D diffuse X-ray scattering, shown in Fig. 1 ▸(*a*), and corresponding 3D-ΔPDF of bixbyite are results of lamellar braunite intergrowths in the bixbyite matrix, which were previously observed by TEM analysis of naturally occurring bixbyite crystals (Peter *et al.*, 2021 ▸; Devilliers & Buseck, 1989 ▸). We expect that the similarity of the bixbyite 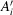 layers and the braunite *A_i_* layers allows the epitaxial intergrowth. Thus, we want to set up a model for the intergrowth structure, from which we can simulate the SCXRS, which may then be directly compared with the experiment. In the following we assume that the approximate relations *a*_braunite_ = *a*_bixbyite_ and *c*_braunite_ = 2*c*_bixbyite_ hold exactly, and that the bixbyite structure is that determined by Roth *et al.* (2018 ▸) at 300 K, *i.e.* we ignore the additional cation site near the 24*d* site for now. For simplicity the metal ions (Mn and Fe) are all assumed to be Fe. The ideal bixbyite structure in our notation can be written as:

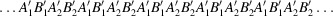

Considering the similarity between the 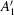 and *A*_1_ layers, we assume that the subsequent layer may be either a 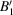 (continuing the bixbyite structure) or a *B*_1_ (seeding a braunite intergrowth). In the case where a braunite layer was seeded, we have to decide for how long the braunite stacking sequence continues, until the bixbyite stacking sequence is resumed. In a recent TEM study, it was reported that most braunite intergrowths had an approximate thickness of 19 Å corresponding to the length of the *c* axis of the braunite unit cell (Peter *et al.*, 2021 ▸). However, considering the similarity of the 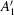 and *A*_1_ layers, it is difficult to distinguish braunite from the bixbyite matrix in the epitaxial interface, using TEM analysis or our modelling. Thus, for our model there is a degree of freedom in choosing the thickness of the braunite intergrowth as long as it is around one *c*_braunite_. An additional point to consider is that the average structure determined by X-ray diffraction is reasonably well described by a model corresponding to the bixbyite structure, *i.e.* the Fe occupancy of the new metal ion site is low with a refined occupancy of 0.070 (7). This indicates that the braunite intergrowths constitute only a small fraction of the overall structure. Furthermore, for simplicity we will assume that the bixbyite matrix is coherent throughout the structure, *i.e.* no stacking faults are introduced. Choosing the braunite intergrowth thickness to be 4*B_i_* and 3*A_i_* fulfils the latter criterion as illustrated by the stacking sequence below (braunite layers are indicated by bold type):

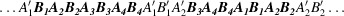

These intergrowths will be 16–17 Å thick. Replacing the 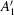 following the first braunite intergrowth by an *A*_1_ layer would result in a intergrowth with a thickness of *c*_braunite_. However, the scattering from such a model is practically indistinguishable from the present choice due to the high similarity of the *A*_1_ and 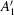 layers.

**Figure 3:**
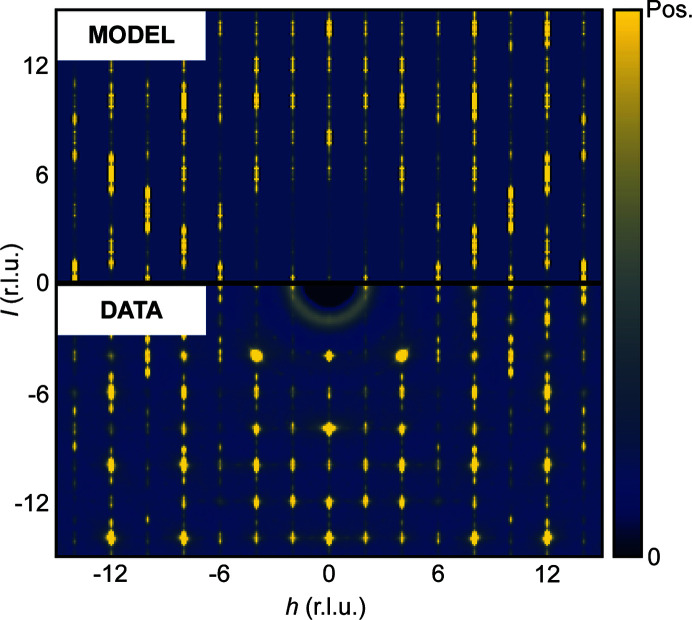
Experimental (bottom) and modelled (top) X-ray diffuse scattering from natural bixbyite single crystal in the *h*0*l* plane of reciprocal space. The experimental data have been convoluted with a Gaussian function with σ = 0.5 pixels to ease comparison with our model data, which are broadened due to the size of the supercell model. A constant background term has been added to the model data.

In order to model the X-ray diffuse scattering, a 10 × 10 × 400 bixbyite supercell was constructed in which any 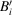 layer had a 2% chance of being replaced by the corresponding *B_i_* layer (with the constraint that braunite intergrowths did not overlap), thereby seeding a braunite intergrowth with a thickness of 16–17 Å. The supercell was then split into forty 10 × 10 × 10 supercells, for which average structure unit cells were computed (the average structure of our supercells deviates from the ideal bixbyite structure, for example by the presence of silicon, due to the inclusion of braunite layers). For each supercell, the single-crystal X-ray scattering was computed on a 501 × 501 × 501 voxel grid spanning ±25 in *h*, *k* and *l* using the *SCATTY* code (Paddison, 2019 ▸). The scattering from all supercells was averaged (the scattering was summed incoherently) and the Bragg scattering was punched and filled using the same procedure as for the experimental data. The isolated diffuse scattering is shown along with the reconstructed experimental single-crystal X-ray diffuse scattering in Fig. 3 ▸.

As seen in Fig. 3 ▸ the supercell model reproduces the experimental diffuse scattering nicely. However, there are some discrepancies. First, a large isotropic background contribution from air scattering is observed in the experimental data. Second, additional diffuse scattering features near Bragg positions are observed. These are attributed to TDS, which peaks at the positions of the Bragg peaks (Willis & Pryor, 1975 ▸).

To be able to better compare the modelled and experimental data, we Fourier transform the diffuse scattering data, thereby obtaining the 3D-ΔPDF. Though the information content is the same, this function makes it easier to compare the experimental and model data, as it is a direct space function with an intuitively simple interpretation in terms of correlations. That is, atomic correlations which have not been modelled can be identified by directly comparing the model 3D-ΔPDF to the experimental counterpart. However, the TDS contribution must still be accounted for, since this will overlap with the 3D-ΔPDF signal arising from braunite intergrowths in some cases. The contribution of TDS was emulated by assuming the atomic displacement correlations to have an isotropic exponentially decaying dependence on the interatomic vector. Such a smooth decay may be expected in materials without significant soft mode dynamics, as exemplified by *e.g.* KCl (Holm *et al.*, 2021 ▸, 2020 ▸). The 3D-ΔPDF for this model was calculated using *YELL* (Simonov *et al.*, 2014 ▸). Details can be found in the supporting information along with the individual 3D-ΔPDFs corresponding to the braunite intergrowth diffuse scattering and the TDS. The overall model 3D-ΔPDF was constructed as a weighted sum of the signal from the intergrowth disorder and TDS – the scale factor is varied manually to approximately match the experimental 3D-ΔPDF.

**Figure 4:**
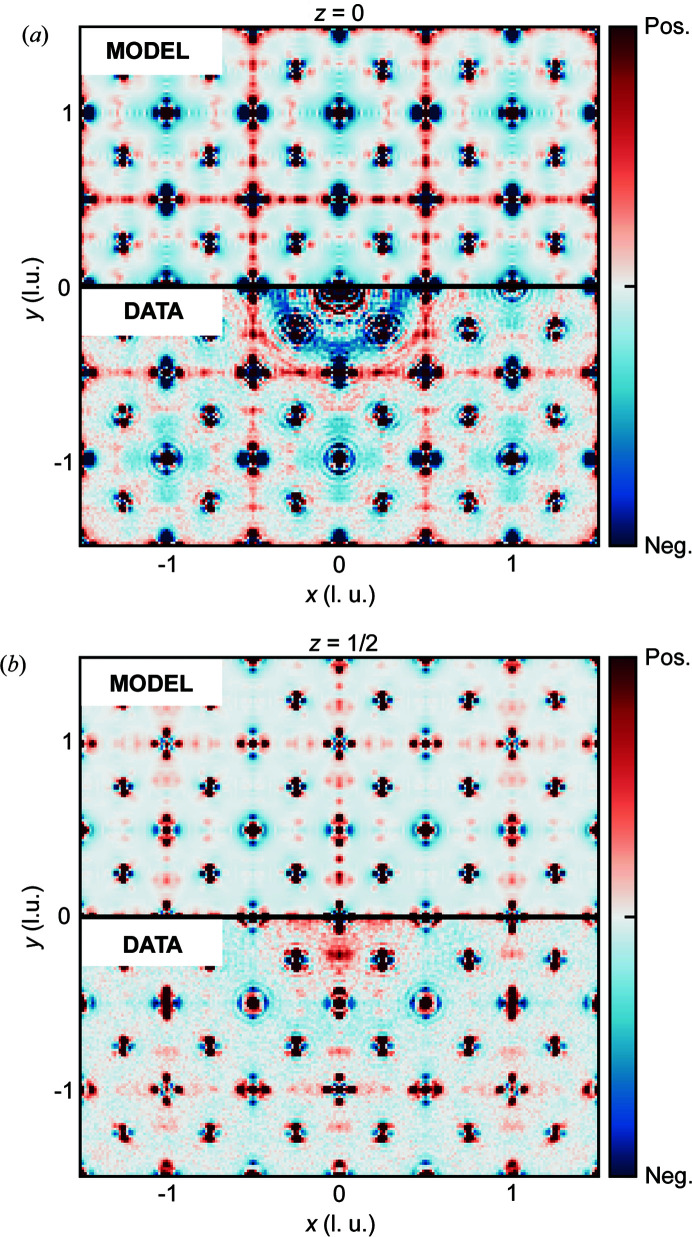
Modelled and experimental 3D-ΔPDFs in the (*a*) *z* = 0 plane and (*b*) *z* = 1/2 plane (fractional coordinates of the bixbyite unit cell). The data are shown on an arbitrary scale.

Slices through the resulting model 3D-ΔPDF can be seen in Figs. 4 ▸(*a*)–4(*b*) for two different values of *z* along with the experimental 3D-ΔPDF. As can be seen, the combined model, including both correlated atomic displacements and the braunite intergrowth model, reproduces the features of the experimental 3D-ΔPDF very well. Thus, the one-dimensional Laue symmetry breaking diffuse scattering observed in our single-crystal X-ray scattering experiments may be described as arising from the epitaxial braunite intergrowths. Note that the intergrowth model diffuse scattering has been multiplied by a Gaussian before Fourier transformation. This emulates the broadening effect of thermal motion in the resulting 3D-ΔPDF. Though our model of bixbyite/braunite intergrowths reproduces the one-dimensional X-ray diffuse scattering, it should be noted that previous TEM studies (Peter *et al.*, 2021 ▸; Kleebe & Lauterbach, 2008 ▸) revealed that natural bixbyite crystals from the Thomas Range, Utah, USA, contain additional impurities to braunite in the form of nanocrystalline jacobsite precipitates. Peter *et al.* (2021 ▸) showed that these precipitates formed in contact with the braunite lamellae although with random orientations and no epitaxial relationship to the host crystal. No clear signature of these precipitates can be found in our diffuse scattering data and we have not included jacobsite in our modelling. This also highlights a case where the local sensitivity of electron microscopy offers information not readily attainable by X-ray or neutron scattering.

**Figure 5:**
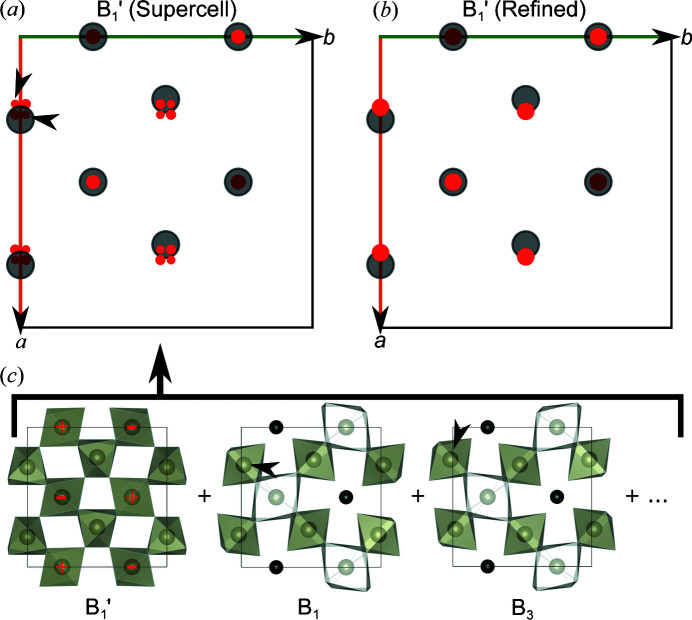
Comparison of the 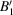 layers of (*a*) the condensed supercell and (*b*) the structure refined against Bragg diffraction (oxide ions have been omitted and 24*d* cation sites are shown in semitransparent grey). The red markers in the 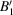 layer of the refined structure indicate the refined positions of the new metal ion site near the 24*d* site. The red markers in the 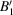 layer of the condensed supercell indicate positions of additional electron density contributed by the cations of the braunite intergrowths. (*c*) Illustrates how the 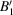 layer of the condensed supercell is obtained by averaging contributions from the bixbyite host crystal and the braunite intergrowths. In the 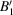 layer in (*c*), red + symbols indicate cations that are displaced out of the *z* = 0.25 plane, and – symbols indicate cations displaced to lower *z* values. In the *B_i_* layers in (*c*), black arrowheads mark examples of cations which contribute to the electron density residuals marked by black arrowheads in the 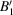 layer of the condensed supercell in (*a*).

As noted earlier, our average structure deviates from that determined in other studies by an additional cation site displaced slightly away from the original 24*d* site. This new metal ion site is located at [0.253 (3), 0, 1/4] and symmetry equivalent positions with a refined Fe occupancy of 0.070 (7). In order to rationalize why additional electron density is observed at this site, we consider the average structure of the supercell model used to simulate the diffuse scattering arising from braunite intergrowths. The average supercell unit cell is computed by averaging the entire 10 × 10 × 400 supercell into one single bixbyite unit cell. A comparison of the 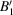 layers of the refined structure and the condensed supercell average structure is given in Figs. 5 ▸(*a*) and 5(*b*).

As seen from Fig. 5 ▸(*b*) the new metal ion site in the refined structure (red markers) corresponds to positions in the supercell average structure where additional electron density is introduced in the supercell average due to the braunite intergrowths shown by red markers in Fig. 5 ▸(*a*). In the 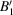 layer the new cation sites are displaced towards the (1/4, 0, 1/4) site (and symmetry equivalents) which is away from the original (0.28463 (2), 0, 1/4) 24*d* site of bixbyite. This can be rationalized by the braunite intergrowths as *e.g.* the *B*_1_ layer of braunite contains Mn at the (0.26739, 0.01739, 1/8) site, whereas the *B*_3_ layer contains Mn at (0.23180, 0.9818, 5/8). Both atoms are indicated by black arrowheads in Fig. 5 ▸(*c*) which shows the bixbyite 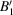 layer along with the braunite *B*_1_ and *B*_3_ layers. When averaged over the entire supercell this gives rise to part of the fourfold partially occupied sites as indicated by the black arrowheads in Fig. 5 ▸(*a*). The remaining additional electron density features can be rationalized in a similar way by considering all the *B_i_* layers of braunite. Additional electron density was also observed in the supercell average near the 8*a* site, which is not symmetry equivalent to the new cation site position near the 24*d* site. However, residuals *are* found near these positions close to the 8*a* site in the average structure refinement, although we are unable to obtain a stable refinement with atoms placed in these positions. These residuals can be seen in the Fourier difference map available in the supporting information. Note that the supercell average has not been symmetry-averaged and therefore different features are observed in positions of the bixbyite unit cell which should be equivalent due to cubic symmetry. In relation to this, note that the average structure data reduction, solution and refinement were performed assuming the cubic *Ia* 3 space group of bixbyite. Though this space group is correct for ideal synthetic bixbyite, it has been shown here that it is only an approximation in the case of natural bixbyite crystals containing one-dimensional disorder due to braunite intergrowths.

From previous single-crystal neutron scattering experiments, it was found that, in order to model the Bragg diffraction data, one had to include the [1, 0, 0; 0, 0, 1; 0, 1, 0] twin law (Roth *et al.*, 2019 ▸, 2018 ▸). This is interesting, since this operation corresponds to a mirror operation interchanging *y* and *z*. Because of the cubic symmetry, this could be equally well described as a twin law interchanging *x* and *y*. The isomorphic relations between the *A*_2_ and *A*_4_ layers of braunite on the one hand and the 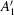 (or 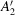 ) layer on the other hand consist of a mirror operation interchanging *x* and *y* and a translation. The mirror twin could therefore result from a braunite intergrowth terminating at a *B*_1_, *B*_3_, *A*_2_ or *A*_4_ layer, since the continuation of the structure would then require the subsequent bixbyite layers to be mirrored with respect to the bixbyite layers preceding the braunite intergrowth. However, this structural feature seems less likely to occur as the periodic bixbyite structure continues to grow, which is the structure our supercell model simulates, since we do not observe the twin formation in our single-crystal X-ray diffraction data. This could be explained by the braunite intergrowths having a finite extent across the bixbyite *ab* plane, resulting in the growth of untwinned bixbyite at the edges of the braunite layer. If the bixbyite nucleation initiates at these bixbyite edges, it is likely that it would be most favourable to form the untwinned bixbyite. Thus, a large single crystal, such as those used for single-crystal neutron scattering, is needed in order to observe the mirror twin.

### 3.3. Mn/Fe—O size effect disorder

The SCNS data shown in Fig. 1 ▸(*b*) contain broad diffuse scattering, which must arise from structural disorder, since it does not display the characteristic decay in reciprocal space associated with magnetic scattering (Squires, 2012 ▸). As our neutron scattering data have a limited completeness at high |*Q*|, it is unsuitable for Fourier transformation, which would otherwise yield an intuitive map similar to the X-ray 3D-ΔPDF. We therefore restrict the analysis to reciprocal space and direct modelling of the diffuse scattering.

A first guess on the short-range correlations giving rise to the diffuse scattering could be correlations in the occupational disorder on Mn/Fe 8*a* and 24*d* sites. This would explain why the disorder mode gives rise to no observable signal in the X-ray data since the scattering powers of Mn and Fe are very similar due to the almost equal number of electrons. However, various different models were attempted in Monte Carlo simulations of cation distributions, but none were able to reproduce the experimental scattering. Thus, it was hypothesized that a dependence of the Mn/Fe—O bond length on the identity of the metal ion could be responsible for the short-range correlations giving rise to the diffuse scattering. In order to test this, a random distribution of Mn and Fe was assumed (with the restriction that the average structure occupancies were fixed for the 8*a* and 24*d* sites) in a 5 × 5 × 5 bixbyite supercell, and the oxide ions were displaced along the Fe/Mn—O interatomic vectors. One could expect that an Mn—O bond would be longer than an Fe—O bond due to size considerations based on the effective nuclear charges of the metal ions. However, this produces diffuse scattering, which is inconsistent with the experimental data. Rather, it was found that the oxide ions are mostly displaced away from Fe and towards Mn.

First, we will explain the model which was found to be consistent with the experimental data, and then we will rationalize the observations based on crystal-field theory. An oxide ion has four neighbours, so we assume that the overall displacement, Δ**r**, is the sum of four individual displacement vectors, δ**r** *_i_* (*i* = 1,…,4). In our model, the individual displacement vectors are chosen to be proportional to the oxide–metal interatomic vector, 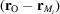 . *M_i_* denotes the type of *i*th neighbouring cation in the supercell which can be either Fe or Mn. The proportionality factor which relates δ**r** *_i_* to 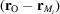 is chosen to be the product of the following parameters. A common model parameter, *x*, determines the ratio of the displacement magnitude relative to length of the interatomic vector. However, an additional parameter, 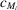 , is introduced to allow the displacement magnitudes for the axial oxide ligands of the 24*d* site, see Fig. 7(*c*), to differ from that of the equatorial ligands and those surrounding the 8*a* site cation. Thus, the value of 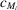 is determined by whether the oxide ion under consideration is an axial ligand to the *i*th metal neighbour, *M_i_*. The sign of the 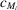 parameter can be negative or positive to allow the displacement of the axial ligands to be in the same or opposite direction to that of the equatorial ligands. 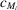 assumes a value of one whenever the oxide ion under consideration is an equatorial ligand to *M_i_* or *M_i_* resides in an 8*a* site. The parameter 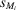 simply determines the sign of the displacement (which can modified by 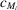 for axial ligands) depending on the identity of the *i*th metal ion neighbour, *M_i_*. Finally, an additional parameter is included in the model. This is the site occupancy factor on the *i*th metal site of the ion which occupies this site in the supercell. The displacement is multiplied by one minus the site occupancy factor 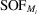 . This factor serves to renormalize the displacement in order to keep the average oxide site position of the supercell to that of the structure refined against the Bragg scattering. The effect can be understood as follows. The 8*a* site has an Mn occupancy of 0.241 (5) and an Fe occupancy of 1–0.241 (5). In order to preserve the average oxide position of a given site all displacements resulting from a neighbouring cation site (that is, δ**r** *_i_* for a fixed oxide site in the average unit cell and fixed *i*) must cancel. Since the 8*a* site is mainly occupied by Fe the direction of the resulting oxide displacements will mainly be that determined by *s*_Fe_, and therefore the displacements for a given oxide site will not cancel when the supercell is averaged to a single unit cell. By renormalizing the displacements by one minus the SOF, the effect of this unequal population is mitigated resulting in the displacement cancelling when averaged over a large enough supercell. Thus, the expression for the displacement of a given oxide ion in the supercell may be cast in the following form:

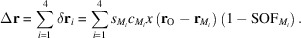

For our final model, the following parameters were found to reproduce the experimental scattering reasonably well:

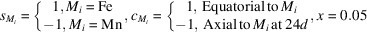

**Figure 6:**
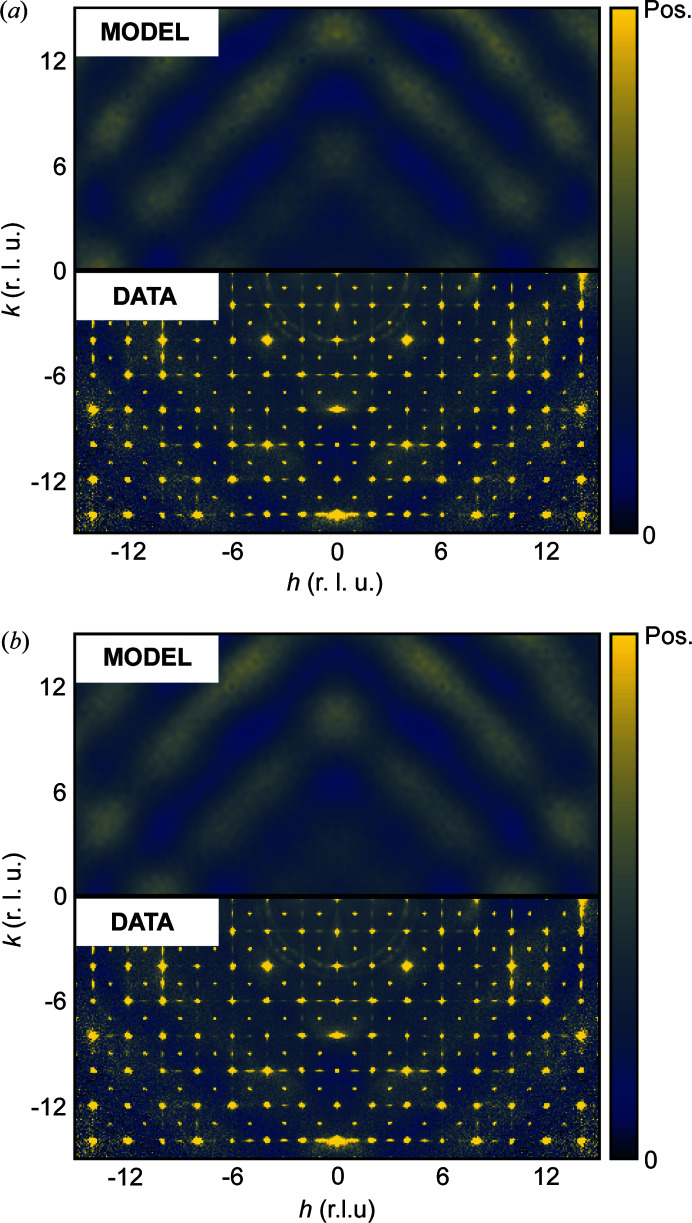
Experimental and modelled single-crystal neutron diffuse scattering data for (*a*) a model assuming oxide displacing away from Fe and towards Mn with the opposite sign of displacements for the axial oxides in the distorted 24*d* site octahedron and (*b*) a model where all the signs of the oxide displacements have been inverted with respect to (*a*).

The corresponding diffuse scattering data in the *hk*0 plane are shown in Fig. 6 ▸(*a*) along with the experimental data. The model data were simulated with *SCATTY* (Paddison, 2019 ▸) using 30 supercells. The supercells were constructed assuming periodic boundary conditions. Data are also shown where the signs of 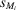 have been swapped in Fig. 6 ▸(*b*) corresponding to the opposite trend in displacements compared with our best model. Simulated diffuse scattering for additional parameter combinations is shown in the supporting information to illustrate the effects on the diffuse scattering.

Note that it would have been ideal if one could determine the discrete possible oxide ion positions by analyzing the Fourier difference maps. However, it was not possible to assign oxide positions based on the residual density features. This could be a result of the presence of braunite intergrowths which can lead to oxide displacements in the braunite–bixbyite interface. Thus, the *ad hoc* displacement model described above was used.

As can be seen from Fig. 6 ▸(*a*), the broad diffuse scattering features of the experimental neutron scattering data are reproduced by our model assuming displacement away from Fe and towards Mn with an opposite sign of displacement for the axial oxides in the distorted 24*d* site octahedron. The model assuming the opposite displacement pattern clearly results in diffuse scattering in positions of reciprocal space where none is observed experimentally, see Fig. 6 ▸(*b*). Though a complete quantification of the oxide displacements should be possible using reverse Monte Carlo methods, this has not been attempted, since this would require isolating the relevant diffuse scattering signal from the braunite intergrowth scattering and the paramagnetic background.

**Figure 7:**
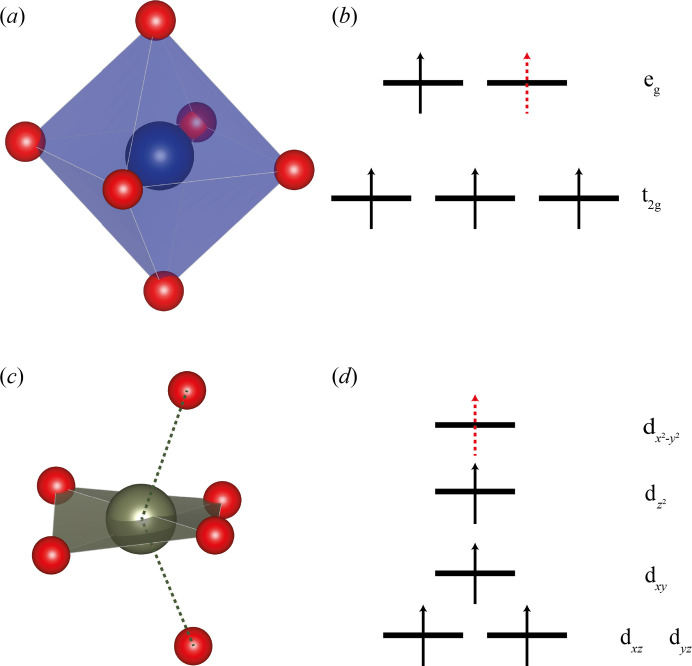
(*a*) and (*b*) 8*a* site octahedron and the idealized, qualitative orbital diagram for Mn(III) and Fe(III). The dashed red arrow corresponds to the extra electron for Fe(III) compared with Mn(III). (*c*) Pseudo-tetragonally distorted octahedral coordination of the 24*d* site; (*d*) qualitative orbital diagram for the corresponding crystal field splitting. For both orbital diagrams, energy increases in the upward direction.

The displacement away from Fe and towards Mn (formally Fe(III) and Mn(III)) can be qualitatively rationalized based on crystal-field theory arguments. The two sites resemble distorted octahedrons with the 24*d* site significantly more distorted than the 8*a* site. The deviation from octahedral symmetry at 8*a* is solely related to the *L*–*M*–*L* angles which are 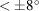 away from the perfect octahedral angle of 90°. Assuming that the crystal-field splitting associated with the 8*a* site can be approximated to that of the perfect octahedral symmetry, see Figs. 7 ▸(*a*)–7(*b*), the first three electrons will occupy the stabilized t_2g_ set resulting in less electrostatic repulsion between the electrons and the ligands and hence allows for a shorter bond length. With the addition of the fourth and fifth electrons (the cases of Mn(III) and Fe(III), respectively) destabilized e_g_ orbitals are occupied, assuming high-spin configuration, resulting in greater electrostatic repulsion between the metal and the ligand and mostly so for Fe(III). Consequently, shorter bond lengths are favoured for Mn(III) compared with Fe(III) as observed from the diffuse scattering.

Switching the focus to the 24*d* site, we expect the same expansion/contraction of the distorted octahedron. However, this site deviates from octahedral symmetry by angles and bond lengths. Specifically, two bonds opposite each other are significantly longer than the remaining four bonds. Let the former correspond to the axial and the latter the equatorial ligands in the distorted octahedron and define a local coordinate system where the *x* and *y* axes lie in the equatorial plane, the best plane through the metal and the four equatorial ligands. From this perspective, the 24*d* site resembles a tetragonally distorted octahedral complex with limited coordination to the axial ligands above and below the *xy* plane, see Fig. 7 ▸(*c*). Assuming the d-orbital splitting corresponds approximately to the perfect tetragonally distorted octahedral splitting, we expect that the Mn(III) axial ligand bond is longer than Fe(III) because the fourth electron occupies the 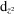 orbital, resulting in more electrostatic repulsion for the axial ligand. With the fifth electron occupying the destabilized 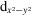 , see Fig. 7 ▸(*d*), there is no energetic gain in elongating the bond to the axial ligand, and we expect the Fe(III) axial bond to be shorter than the Mn(III) axial bond.

In summary, the metal–ligand bonds should compress in Mn(III) and elongate in Fe(III) octahedrons with the exception of the axial bonds of the 24*d* site. By describing the octahedron as having tetragonally distorted octahedral geometry with limited axial ligand coordination, we expect the axial metal–ligand bonds at this site to compress/elongate in the opposite way to the remaining bonds.

This oxide relaxation may play an important role with respect to the presence of the magnetically disordered spin-glass phase observed at low temperatures. The cations in bixbyite form a distorted f.c.c. sublattice. As argued by Roth *et al.* (2019 ▸), one could thus expect the same geometrical frustration in bixbyite as for a perfect f.c.c. lattice, which requires a next-nearest neighbour interaction for it to order antiferromagnetically (Lines, 1965 ▸; Anderson, 1950 ▸; Li, 1951 ▸). Assuming the exchange interaction between nearest neighbour cations to be mediated by super-exchange through the oxide ions, it could be expected that a suitable oxide–metal bond elongation resulting in low orbital overlap could break a next-nearest neighbour interaction. However, establishing such a connection is beyond the scope of the current study, since the distorted environments of the cations and the mixed occupancy complicate a detailed analysis of the super-exchange interaction, combined with the fact that we were unable to assign discrete oxide positions for the displacement model.

## 4. Conclusions

We have shown that the one-dimensional Laue symmetry breaking observed in single-crystal X-ray diffuse scattering from naturally occurring bixbyite crystals from the Thomas Range, Utah, USA, arises from the presence of braunite intergrowths. Analysis of the Bragg diffraction signal showed that the residuals observed in the average structure refinement arise as a result of this one-dimensional disorder. This observation supports the idea of a well defined epitaxial relationship between the braunite and bixbyite structures at the intergrowth interface.

Using single-crystal neutron diffuse scattering, we showed how the oxide ion sublattice relaxes due to the substitutional disorder on the shared Fe/Mn sites. Particularly, it has been shown how the experimental data are consistent with a random distribution of the cations and displacements of the oxide ions which could be rationalized by simple crystal-field theory considerations.

This study highlights the complementarity of neutron and X-ray single-crystal scattering and in particular demonstrates its application to systems exhibiting multiple modes of disorder.

## Supplementary Material

Supporting figures. DOI: 10.1107/S2052252522006315/fc5062sup1.pdf
