# Supplementary material for: Epitaxial intergrowths and local oxide relaxations in natural bixbyite Fe2−x Mn x O3
Source: IUCrJ. 2022 Jun 29;9(Pt 4):523–32. doi: 10.1107/S2052252522006315 (PMC9252160; doi:10.1107/S2052252522006315)
Supplement: Supplementary file 1 [file m-09-00523-sup1.pdf]

IUCrJ

Volume 9 (2022)

Supporting information for article:

Epitaxial intergrowths and local oxide relaxations in natural bixbyite $Fe_{2-x}Mn_xO_3$

Kristoffer A. H. Støckler, Nikolaj Roth, Bjørn B. E. Grønbech and Bo Brummerstedt Iversen

A. Ad-hoc model for correlated atomic displacements

As described in the main text, the contribution of correlated atomic displacements to the 3D-ΔPDF was simulated using YELL [1] assuming a simple model for the correlation. All atom pairs, within the asymmetric cone of the m$\bar{3}$ point group, with difference positions vectors with x-, y-, and z-components between -4.75 and 4.75 (fractional units of lattice parameter) were included. It was assumed that the correlations had an exponential dependence on the interatomic vector, $\exp(-\zeta \cdot |\boldsymbol{r_j} - \boldsymbol{r_i}|)$, with ζ chosen by trial and error to be 0.3. Thus, the correlations are assumed isotropic in PDF space. However, the magnitude of the covariance is modified by the square root of product of the atomic displacement parameters, and is thus not an isotropic decaying function in PDF space. A further simplifying assumption was, that the correlation matrix was diagonal with all diagonal elements equal. This is generally not the case, since the correlation matrix is only restricted by the point group symmetry of the atomic pair. However, the result of this simplification is that the shape of the features in the 3D-ΔPDF become isotropic which does not affect the qualitative comparisons that are made in this study significantly.

B. 3D-ΔPDFs from intergrowth and YELL models

3D-ΔPDFs for the braunite intergrowth model and the YELL model described in the preceding section are shown for z=0 and z=0.5 in Fig. S1. The final model shown in the main text corresponds to adding the two models.

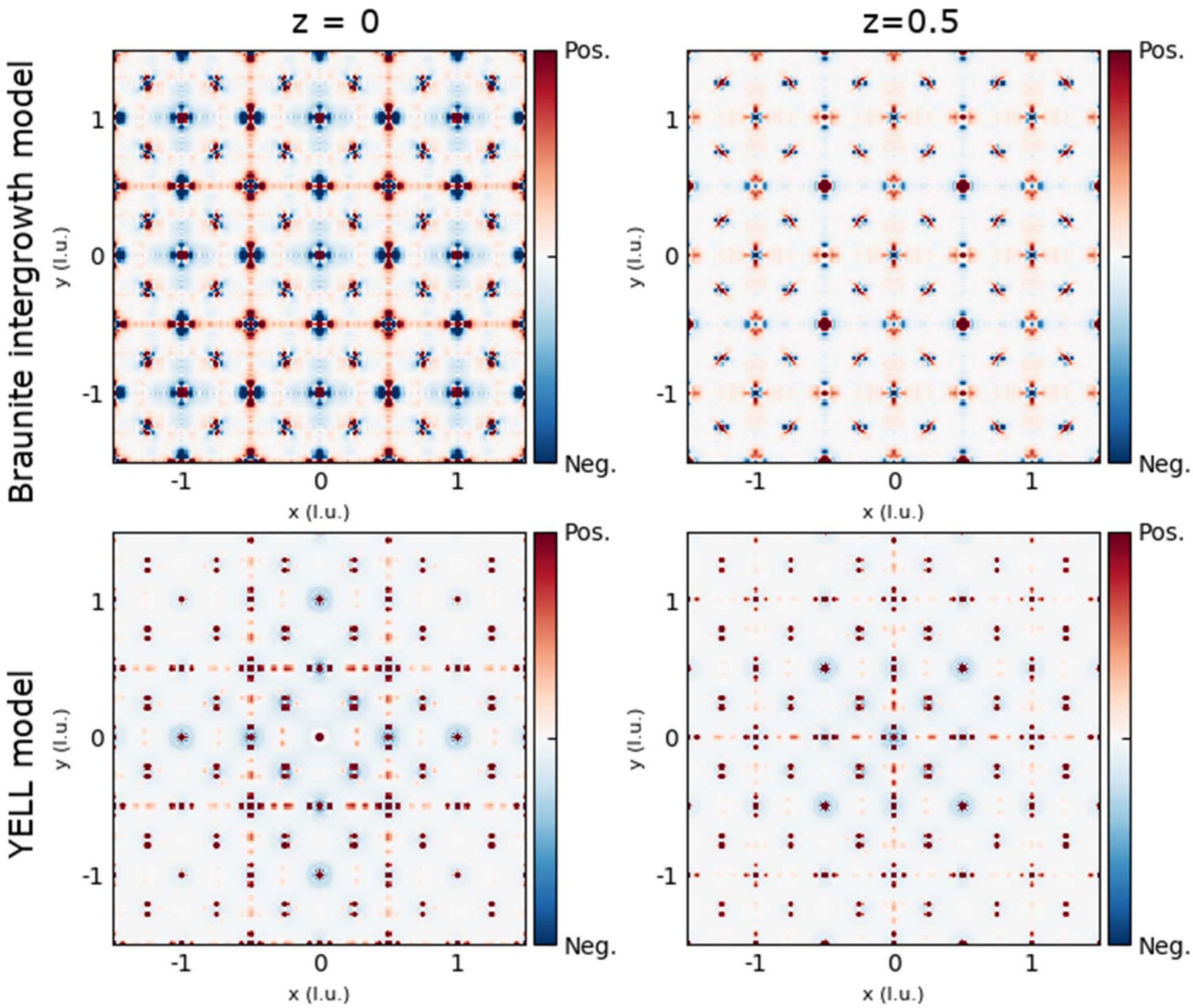

Fig. S1. Slices through the 3D-ΔPDFs for the braunite intergrowth model (top row) and YELL model (bottom row) for z=0 (left column) and z=0.5 (right column). In order to obtain the final model the two components are added (corresponds to incoherent addition of the scattering signals).

C. Fourier difference map in the A'₁-layer

The Fourier difference map of the A'$_1$-layer of the refined Bixbyite structure is given in Fig. S2. As seen from this map, there is additional electron density near the 8a site as anticipated based on the average structure of the supercell model. However compared to the average structure of the supercell model, the residual electron density shows an octahedral structure around the 8a site (there are also residuals

pointing out of the *ab*-plane not shown in Fig. S2). This is expected to be caused by the assumption of cubic symmetry in the treatment of the Bragg diffraction data.

Furthermore, we also observe additional residual density near the 24d site. In the supercell average structure, the electron density contribution of the braunite intergrowth near the 24d site of bixbyite is split into several sites. Thus, the description used in the average structure refinement with only one additional Fe-site is insufficient for describing the entirety of the braunite electron density contribution.

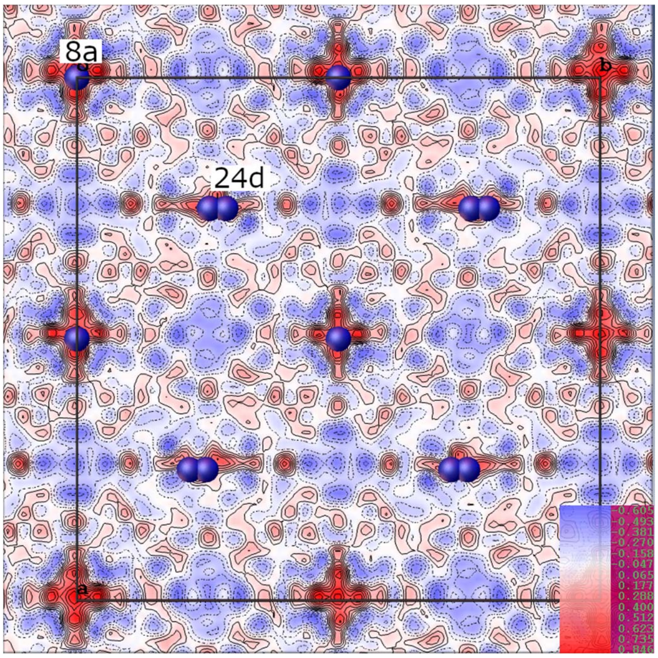

Fig. S2. Fourier difference map in the A'$_1$-layer of the refined Bixbyite structure. Red denotes electron density not described by the model, while blue denotes regions in which the model describes more electron density compared to what is described by the data. The blue spheres denote positions of Fe. The Fourier difference map should be compared to Fig. 5 in the main text.

D. Model neutron scattering for additional parameter combinations

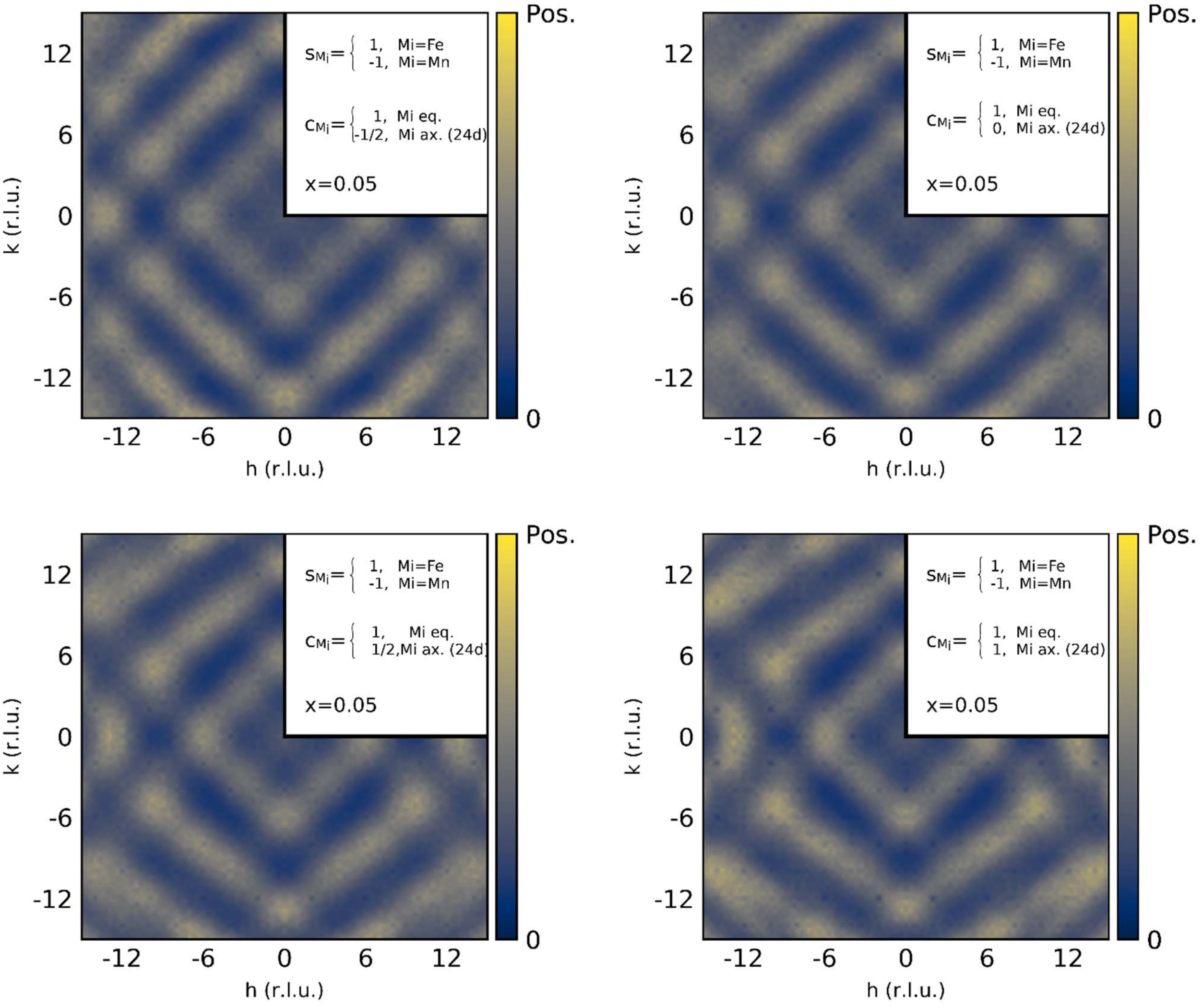

Fig. S3. Model neutron scattering in the hk0-plane for various values of c_{M_i}. All parameters are listed in the individual subfigures. Compared to the values found to reproduce the experimental data the best, varying c_{M_i} distorts the reciprocal space distribution of the diffuse scattering.

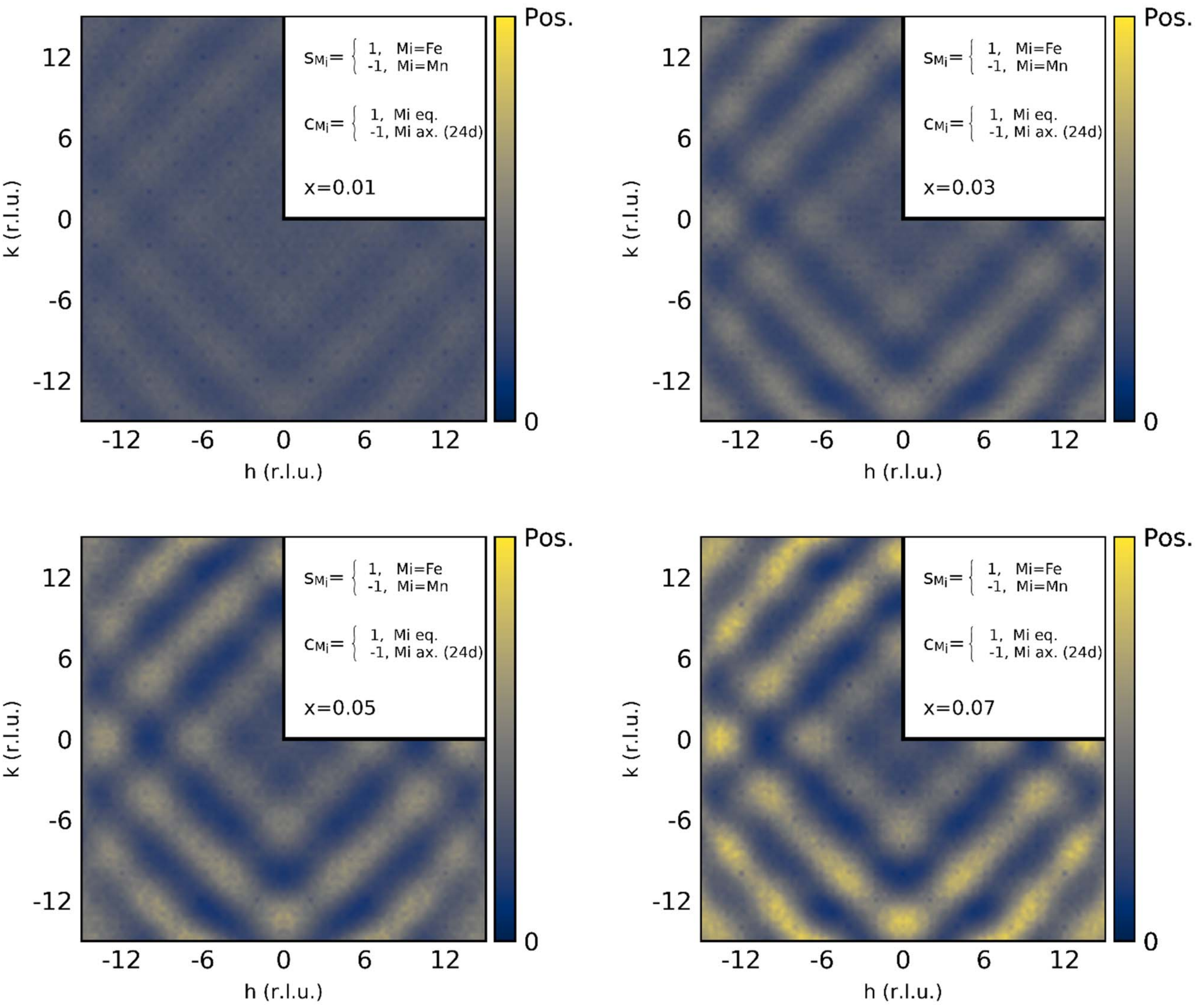

Fig. S4. Model neutron scattering in the hk0-plane for various values of x. All parameters are listed in the individual subfigures. A larger value of x (corresponding to larger oxide displacements) leads to a higher intensity of the diffuse scattering features.

E. References

[1] A. Simonov, T. Weber, and W. Steurer, J Appl Crystallogr **47**, 1146 (2014).
